# Hepatic NLRP3-Derived Hsp70 Binding to TLR4 Mediates MASLD to MASH Progression upon Inhibition of PP2A by Harmful Algal Bloom Toxin Microcystin, a Second Hit

**DOI:** 10.3390/ijms242216354

**Published:** 2023-11-15

**Authors:** Subhajit Roy, Punnag Saha, Dipro Bose, Ayushi Trivedi, Madhura More, Shuo Xiao, Anna Mae Diehl, Saurabh Chatterjee

**Affiliations:** 1 Environmental Health and Disease Laboratory, Department of Environmental and Occupational Health, Program in Public Health, Susan and Henry Samueli College of Health Sciences, University of California, Irvine, CA 92697, USA; subhajr@uci.edu (S.R.); punnags@uci.edu (P.S.); diprob@uci.edu (D.B.); aktrived@uci.edu (A.T.);; 2 Department of Pharmacology and Toxicology, Ernest Mario School of Pharmacy, Rutgers University, Piscataway, NJ 08854, USA; sx106@pharmacy.rutgers.edu; 3 Division of Gastroenterology, Department of Medicine, Duke University, Durham, NC 27710, USA; diehl004@mc.duke.edu; 4 Division of Infectious Diseases, School of Medicine, University of California, Irvine, CA 92697, USA

**Keywords:** MASLD, microcystin, inflammasome, NLRP3, necroptosis, heat shock protein

## Abstract

Harmful algal bloom toxin microcystin has been associated with metabolic dysfunction-associated steatotic liver disease (MASLD) progression and hepatocellular carcinoma, though the mechanisms remain unclear. Using an established mouse model of MASLD, we show that the NLRP3–Hsp70–TLR4 axis drives in part the inflammation of the liver lobule that results in the progression of MASLD to metabolic dysfunction-associated steatohepatitis (MASH). Results showed that mice deficient in NLRP3 exhibited decreased MASH pathology, blocked Hsp70 expression, and co-binding with NLRP3, a crucial protein component of the liver inflammasome. Hsp70, both in the liver lobule and extracellularly released in the liver vasculature, acted as a ligand to TLR4 in the liver, primarily in hepatocytes to activate the NF-κB pathway, ultimately leading to hepatic cell death and necroptosis, a crucial pathology of MASH progression. The above studies show a novel insight into an inflammasome-triggered Hsp70-mediated inflammation that may have broader implications in MASLD pathology. MASLD to MASH progression often requires multiple hits. One of the mediators of progressive MASLD is environmental toxins. In this research report, we show for the first time a novel mechanism where microcystin-LR, an environmental toxin, advances MASLD to MASH by triggering the release of Hsp70 as a DAMP to activate TLR4-induced inflammation in the liver.

## 1. Introduction

Globally, the spectrum of metabolic dysfunction-associated steatotic liver disease (MASLD) related illness is steadily rising; about 38% of the adult population suffers from it [1,2,3]. MASLD has emerged as a significant public health concern due to its high prevalence and strong association with environmental and genetic factors [4,5,6,7]. The progression of nonalcoholic steatohepatitis (NASH), now known as metabolic dysfunction-associated steatohepatitis (MASH), follows a multi-hit hypothesis, where exposure to environmental contaminants acts as a secondary hit, exacerbating MASLD and promoting its progression to MASH and liver fibrosis [6,8]. The progression involves a complex interplay of various mechanisms. Initially, either leptin resistance or insulin resistance leads to fat accumulation in the liver (steatosis), and the subsequent hit involves oxidative stress, Kupffer cell activation, proinflammatory responses, and fibrogenesis. The multiple parallel hits hypothesis suggests that oxidative stress, inflammation, lipotoxicity, and insulin resistance are key contributors. Hepatocellular injury is triggered by oxidative stress, resulting from an imbalance between reactive oxygen species and antioxidants [9,10,11,12]. Inflammation is driven by immune cells and proinflammatory cytokines, contributing to further liver damage, and lipotoxicity occurs due to the toxic accumulation of lipids in liver cells, leading to dysfunction and cell death [13,14]. Insulin resistance worsens these processes, promoting lipid buildup, inflammation, and fibrosis [15].

Epidemiological studies have shown that exposure to harmful algal blooms and the concomitant release of microcystins are strongly associated with hepatocellular carcinoma and MASLD development [16,17]. Recent cyanobacterial blooms have raised concerns as they release protein phosphatase 2A (PP2A) inhibitors like microcystin-LR (MC-LR), posing a significant threat to individuals with MASLD [18]. Mechanistically, MC-LR acts as a protein phosphatase inhibitor, particularly targeting Ser/Thr protein phosphatase 1 (PP1) and protein phosphatase 2A (PP2A) by inhibiting the catalytic activities of the enzymes [19,20]. PP2A is a family of Ser/Thr phosphatases composed of the catalytic C subunit and regulatory A subunit that is often associated with B-type subunits [21]. The precise role of B-type subunits in controlling pathological processes in the liver remains elusive [22]. Liver-specific knockouts of the PP2A gene have demonstrated decreased lipid deposition (steatosis) [23]. Inhibiting PP2A and decreasing steatosis may provide insights into the role of PP2A in MASLD, particularly in the presence of environmental factors like MC-LR. Primarily, PP2A is responsible for maintaining cellular homeostasis, including regulating the cell cycle, cellular development, tumor suppression, and signal transduction [22]. MC-LR binds covalently to the catalytic subunit of PP2A, leading to a decrease in its catalytic activity [21]. The diminished catalytic activity of PP2A disrupts normal cellular activities and signaling pathways. Previous studies have elucidated the role of the PP2A inhibitor, MC-LR, in inducing oxidative stress, ER stress, autophagy, apoptosis, pyroptosis, and necroptosis in the context of hepatic tissue injury, cell death, and survival decisions [24]. In the context of MASLD, inhibiting PP2A is associated with worsening of liver injury. Recent studies examined the protective role of PP2A in liver pathology and its correlation with disease progression [25]. Interestingly, the roles of PP2A inhibition are diverse, with some studies showing that PP2A inhibitors like okadaic acid can restore AMPK phosphorylation in alcoholic fatty liver disease [26], while inhibitors like Imipramine and LB100 attenuate liver injury in both alcoholic and non-alcoholic liver diseases [27,28]. Thus, further studies that focus on the mechanisms are needed to explore the role of exogenous PP2A inhibitors such as MC-LR.

In our previous studies, we observed that the activation of nucleotide-binding domain-like receptor protein 3 (NLRP3) plays a significant role in triggering the inflammatory responses in the MASLD condition upon exposure to the PP2A inhibitor, MC-LR [29]. The NLRP3 inflammasome comprises various proteins located within a single complex [30]. It consists of the cytoplasmic receptor NLRP3, acting as the sensor molecule for the inflammasome, the adaptor protein ASC2 (caspase-recruitment domain apoptosis-associated speck-like protein containing a CARD 2), and the effector protein pro-caspase-1. In the activated stage, these components assemble and bring multiple pro-caspase subunits into proximity. Subsequently, the pro-caspase-1 molecules within the cytosolic multimolecular complexes cleave themselves, releasing the mature or active form of caspase-1. This activated caspase-1 further cleaves pro-interleukin-1β (pro-IL-1β) and pro-interleukin-18 (pro-IL-18) into their mature interleukin forms IL-1β and IL-18, which are then released into the system [27,28,29].

The role of heat shock proteins (HSPs) in immune responses, inflammatory events, and immunomodulation has been extensively investigated [31,32]. These proteins have shown their ability to modulate various inflammasomes, including NLRP3 [33]. The significance of HSPs has also been explored in the context of non-alcoholic fatty liver disease (MASLD) and protein phosphatase 2A (PP2A) inhibition [34,35,36]. Among them, heat shock protein 70 (Hsp70) has emerged as a crucial player in antigen presentation, T-cell priming, and immune response [37,38,39]. It is worth noting that Hsp70 exists in two distinct forms, intracellular and extracellular [40]. Intracellular Hsp70 has been reported to regulate inflammation, while the extracellular form exhibits inflammatory properties [41]. When released into the extracellular environment, Hsp70 acts as a Damage Associated Molecular Pattern (DAMP), triggering inflammatory responses [42,43]. The function of Hsp70 as a toll-like receptor 4 (TLR4) ligand has been well-documented in various studies [44,45]. TLR4 is known for its role in activating the nuclear factor kappa-light-chain-enhancer of activated B cells (NF-κB) pathway, which involves the phosphorylation and nuclear import of the active NF-κB complex, leading to enhanced transcription of genes related to immune response and inflammation [42,46]. In hepatocytes, the functional attributes of TLR4 have been extensively studied in models of hepatic injury and MASH [47,48]. Importantly, the Hsp70–TLR4 axis has been shown to activate NF-κB downstream [44]. Furthermore, Hsp70 has been shown to physically interact with NLRP3, thereby influencing its activation status [33,41]. Importantly, in the context of MASLD, the anti-inflammatory functions of intracellular Hsp70 seem to be compromised [49]. Additionally, it has been observed that PP2A inhibitors can upregulate the expression of Hsp70 [50] and activates the NF-κB pathway [51]. Importantly, NLRP3 could serve to determine the Hsp70 secretion in various tumors in conferring robust immune response mediated by the NLRP3–Hsp70–TLR4 axis [45,52]. However, the context of Hsp70 production to its release and concomitant mechanisms of extracellular Hsp70 in conferring inflammatory responses remain poorly understood in the context of MASLD and PP2A inhibition following exposures to harmful algal bloom toxins.

Therefore, our study aimed to investigate the NLRP3–Hsp70–TLR4 axis, the mechanistic relationship between NLRP3 and Hsp70–TLR4 interaction in the liver upon PP2A inhibitor exposure with underlying conditions of MASLD, and to validate the findings by using NLRP3 knockout mice and in vitro studies. This study may serve as an important mechanistic insight into the progression of MASLD to MASH and have subsequent implications in various MASH etiologies.

## 2. Results

### 2.1. NLRP3 Knockout (KO) Alleviated the Pathophysiology of MASLD to MASH Progression upon PP2A Inhibition

**Figure 1:**
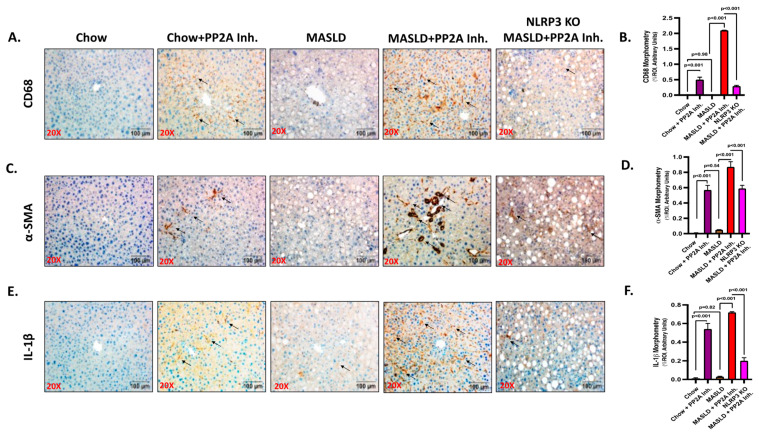
NLRP3 Knockout (KO) alleviates the pathophysiology of MASLD to MASH progression upon PP2A inhibition. (**A**) CD68 immunoreactivity is shown by immunohistochemistry in liver sections from Chow, Chow + PP2A inhibitor, MASLD, MASLD + PP2A inhibitor, and NLRP3 KO MASLD + PP2A inhibitor mouse samples. Images were taken at 20× magnification and displayed with a scale of 100 μm. Some of the immunoreactive sites were indicated by arrows. (**B**) Morphometry of CD68 immunoreactivity in all the groups was measured as arbitrary light units from three separate microscopic fields and plotted along the ordinate. All *p*-values were determined through one-way ANOVA with the significance level set at *p* < 0.05. (**C**) α-SMA immunoreactivity is shown by immunohistochemistry in liver sections from Chow, Chow + PP2A inhibitor, MASLD, MASLD + PP2A inhibitor, and NLRP3 KO MASLD + PP2A inhibitor mouse samples. Images were taken at 20× magnification and displayed with a scale of 100 μm. Some of the immunoreactive sites were indicated by arrows. (**D**) Morphometry of α-SMA immunoreactivity in all the groups was measured as arbitrary light units from three separate microscopic fields and plotted along the ordinate. All *p*-values were determined through one-way ANOVA with the significance level set at *p* < 0.05. (**E**) IL-1β immunoreactivity is shown by immunohistochemistry in liver sections from Chow, Chow + PP2A inhibitor, MASLD, MASLD + PP2A inhibitor, and NLRP3 KO MASLD + PP2A inhibitor mouse samples. Images were taken at 20× magnification and displayed with a scale of 100 μm. Some of the immunoreactive sites were indicated by arrows. (**F**) Morphometry of IL-1β immunoreactivity in all the groups was measured as arbitrary light units from three separate microscopic fields and plotted along the ordinate. All *p*-values were determined through one-way ANOVA with the significance level set at *p* < 0.05.

A comparative analysis revealed significant aggravation of the liver pathophysiology in the MASLD + PP2A inhibitor group when compared to MASLD control or chow-fed mice exposed to the PP2A inhibitor (Chow + PP2A Inhibitor) (Figure 1; *p* < 0.001). Past studies from our lab indicated that activation of the NLRP3 inflammasome causes significant inflammation in the liver and brain tissue of the MASLD/MASH mouse models [7]. In this section, the involvement of the NLRP3 inflammasome in the hepatic inflammation and tissue injury was investigated in terms of PP2A inhibitor-induced exacerbation of the MASLD pathology using a transgenic mice group devoid of the NLRP3 gene (NLRP3 KO MASLD + PP2A inhibitor). Immunohistochemistry was performed on liver tissue sections to assess the expression of Kupffer cell activation marker CD68, fibrotic liver damage marker α-SMA, and proinflammatory cytokine IL-1β. The highest level of hepatic injury was observed in the MASLD + PP2A inhibitor group compared to other groups (Figure 1; *p* < 0.001). The results showed a significant decrease in the amount of Kupffer cell activation (Figure 1A,B; *p* < 0.001), fibrosis (Figure 1C,D; *p* < 0.001), and IL-1β (Figure 1E,F; *p* < 0.001) levels in the NLRP3 KO MASLD + PP2A inhibitor group compared to the highest reactive MASLD + PP2A inhibitor group. More than a five-fold decrease in the CD68 immunoreactivity was observed in the absence of NLRP3 protein (Figure 1A,B; *p* < 0.001), and a similar trend was noted in fibrosis profile and IL-1β release (Figure 1C–F; *p* < 0.001).

### 2.2. NLRP3 Drove the Inflammatory Events and Interacted with Hsp70 in Aggravating the Hepatic Injury

**Figure 2:**
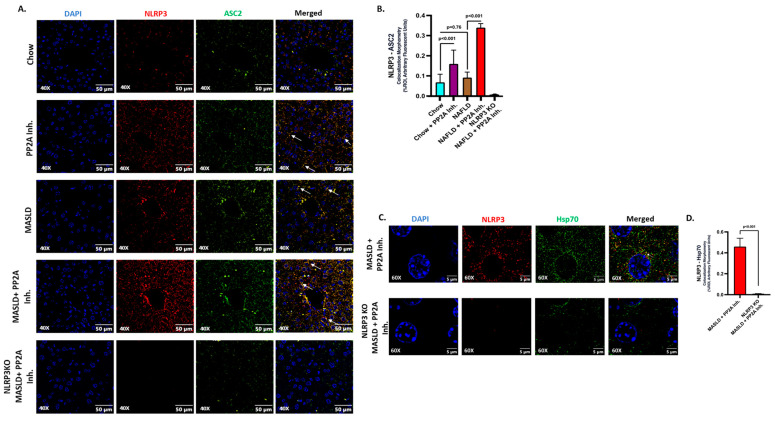
NLRP3 drives the inflammatory events and interacts with Hsp70 in aggravating the hepatic injury. (**A**) Immunofluorescence dual labeling of NLRP3 (red), ASC2 (green), and colocalization (yellow) taken at 40× magnification, displayed with a scale of 50 μm in the liver sections from Chow, Chow + PP2A inhibitor, MASLD, and MASLD + PP2A inhibitor mouse samples. Some of the colocalization events were indicated by white arrows. (**B**) Morphometry of NLRP3–ASC2 colocalization (yellow) events in all the groups were measured as arbitrary fluorescent units from three separate microscopic fields and plotted along the ordinate. All *p*-values were determined through one-way ANOVA with the significance level set at *p* < 0.05. (**C**) Immunofluorescence dual labeling of NLRP3 (red), Hsp70 (green), and colocalization (yellow) taken at 60× magnification, displayed with a scale of 5 μm in the liver sections from MASLD + PP2A inhibitor and NLRP3 KO MASLD + PP2A inhibitor mouse samples. Some of the colocalization events are indicated by white arrows. (**D**) Morphometry of NLRP3–Hsp70 colocalization (yellow) events in both groups were measured as arbitrary fluorescent units from three separate microscopic fields and plotted along the ordinate. All *p*-values were determined through an unpaired *t*-test with the significance level set at *p* < 0.05.

NLRP3 inflammasome activation was implicated in various acute and chronic liver diseases, contributing to the pathologies related to immune cell activation, hepatocyte damage, and events of hepatic inflammation [53,54,55,56]. Past research from our lab has elucidated that PP2A inhibitors cause NLRP3 activation in intestinal and liver tissues [7,29]. In the current study, the role of NLRP3 activation in liver tissues of respective animal groups was demonstrated and correlated with its contribution as the causal factor in determining the pathophysiological outcomes. The representative immunofluorescence images reflect the activation status of NLRP3 measured in terms of NLRP3 (red) and ASC2 (green), and colocalization events (yellow) in DAPI counterstained liver slices; the highest inflammation was observed in the MASLD + PP2A inhibitor group, followed by Chow + PP2A inhibitor group (Figure 2A; *p* < 0.001). The morphometric analysis demonstrated a significant increase in the NLRP3 activation profile in the presence of the PP2A inhibitor, which was further amplified when co-exposed with MASLD (MASLD + PP2A inhibitor group) (Figure 2B; *p* < 0.001).

HSPs are reported to modulate different inflammasomes including NLRP3 [33]. The role of HSPs was investigated before in the context of MASLD and PP2A inhibition [34,35,36]. Hence, several pertinent HSPs were probed in the liver tissues of our respective sample groups, and Hsp70 was selected as the candidate after the screening (*p* < 0.001, Supplementary Figure S1) due to its prominent functional associations with NLRP3 and its potential in contributing to the observed liver pathology in the conditions of MASLD and PP2A inhibition. Hsp70 is known to interact physically with intracellular NLRP3 and regulate its activation status [41]. In the present study, we observed that NLRP3 physically interacts with Hsp70 in liver tissue upon co-exposure to MASLD and PP2A Inh. (Figure 2C,D; *p* < 0.001) as suggested by immunolocalization studies.

### 2.3. NLRP3 Was Key to the Production and Release of Hsp70 in the Liver

The existing literature has reported that NLRP3 possibly serves as an upstream determinant of the Hsp70 secretion in tumor tissues [45,52]. Extracellular Hsp70 is known to act as a DAMP and induce inflammatory responses [42]. The MASLD condition has also been linked to a disrupted intracellular Hsp70 activity which otherwise serves as an anti-inflammatory molecule and PP2A inhibitors are reported to increase the expression of Hsp70 [50]. However, the involvement and characterization of Hsp70 in the conditions of MASLD and PP2A inhibition remained elusive. Hence, further investigation was necessary to mechanistically establish the relationship between NLRP3 and Hsp70 in this context.

**Figure 3:**
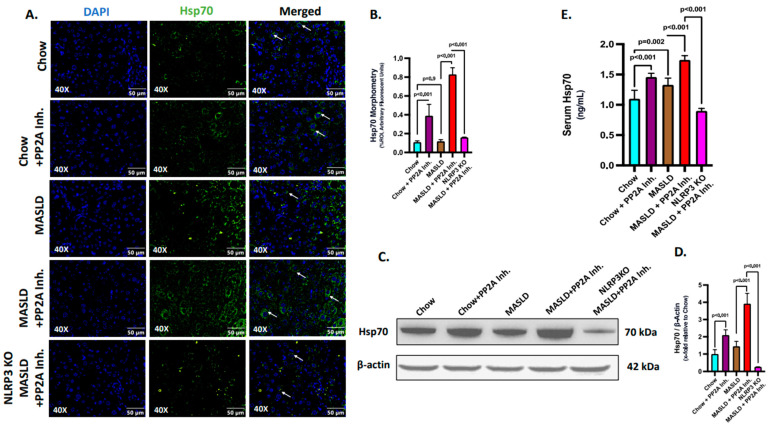
NLRP3 determines the production and release of Hsp70 in the liver. (**A**) Immunofluorescence labeling of Hsp70 (green) taken at 40× magnification, displayed with a scale of 50 μm in the liver sections from Chow, Chow + PP2A inhibitor, MASLD, MASLD + PP2A inhibitor, and NLRP3 KO MASLD + PP2A inhibitor mouse samples. Some of the immunoreactivity events were indicated by white arrows. (**B**) Morphometry of Hsp70 (green) immunoreactivity in all the groups was measured as arbitrary fluorescent units from three separate microscopic fields and plotted along the ordinate. All *p*-values were determined through one-way ANOVA with the significance level set at *p* < 0.05. (**C**) Immunoblot analysis of Hsp70 and β-actin protein level expressions from the liver lysates of Chow, Chow + PP2A inhibitor, MASLD, MASLD + PP2A inhibitor, and NLRP3 KO MASLD + PP2A inhibitor mouse samples. (**D**) Densitometric analysis of Hsp70 reactivity calculated as the ratio of Hsp70 to β-actin, displayed as mean and deviation (*n* = 3 in biological replicates). All *p*-values were determined through one-way ANOVA with the significance level set at *p* < 0.05. (**E**) Serum Hsp70 level in ng/mL was plotted as a bar graph in Chow, Chow + PP2A inhibitor, MASLD, MASLD + PP2A inhibitor, and NLRP3 KO MASLD + PP2A inhibitor mouse serum samples (*n* = 6 in biological replicates). All *p*-values were determined through one-way ANOVA with the significance level set at *p* < 0.05.

Firstly, an initial study was conducted to assess the production and secretion of Hsp70 at the hepatic and systemic levels. The expression profile of Hsp70 was assessed through immunofluorescence (Green, counterstained in DAPI) in the liver sections of the experimental mice groups (Figure 3A,B). We observed that the Hsp70 expression was significantly increased in the MASLD + PP2A inhibitor group, followed by the Chow + PP2A inhibitor group, and a significant decrease in the NLRP3 KO MASLD + PP2A inhibitor group, comparatively (Figure 3A,B; *p* < 0.001). The production of Hsp70 in the liver was also measured by immunoblot assay and a similar trend was observed: the highest expression was observed in the MASLD + PP2A inhibitor group, followed by the Chow + PP2A inhibitor group, and a significant decrease in the NLRP3 KO MASLD + PP2A inhibitor group (Figure 3C,D; *p* < 0.001). Results indicated that NLRP3 markedly deceased the production of Hsp70 in the liver tissue. Further, the secreted form of Hsp70 was measured through ELISA from the serum samples of the experimental mice groups. The results revealed that the serum levels of Hsp70 were increased in both the MASLD (*p* = 0.02) and Chow + PP2A inhibitor (*p* < 0.001) groups, with the highest level observed in the MASLD + PP2A inhibitor group and a significant decrease noted in the NLRP3 KO MASLD + PP2A inhibitor group (Figure 3E; *p* < 0.001). The results suggested that the serum levels of Hsp70 were observed to be under strict modulation by NLRP3 along with its production in liver tissues.

### 2.4. Hsp70 Served as a Ligand of TLR4 to Initiate the Inflammatory Cascade in Hepatocytes

The function of the extracellular Hsp70 as a TLR4 ligand is well documented in earlier studies [44,45]. In this section, further investigations were undertaken to probe the ligand binding activity and initiation of the Hsp70–TLR4 signaling cascade in the liver cells, especially in hepatocytes. The Hsp70 produced and secreted by liver tissue was correlative with its systemic level, and the increased levels of serum Hsp70 in the MASLD + PP2A group when compared to the MASLD-only group also implicated the higher extracellular levels of Hsp70 (Figure 3E; *p* < 0.001). The source may be primarily from the liver but that may be speculation at this point; however, a parallel hepatic Hsp70 level increase together with high circulatory levels of the same protein may be indicative of extracellular Hsp70 acting in a paracrine fashion in the liver microenvironment.

**Figure 4:**
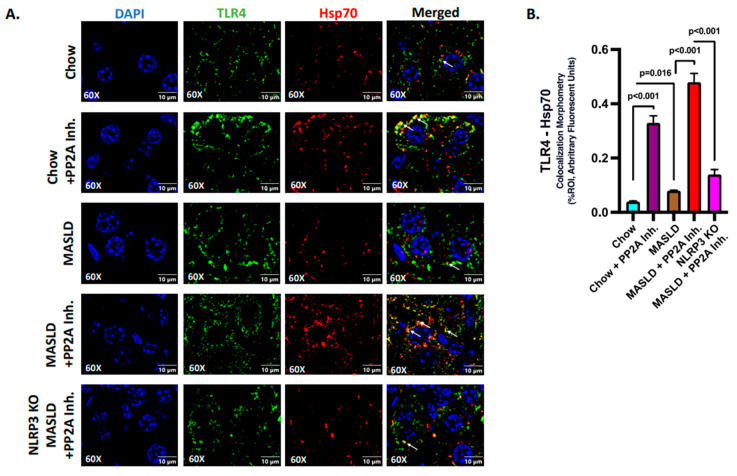
Hsp70 serves as a ligand of TLR4 to initiate the inflammatory cascade in hepatocytes. (**A**) Immunofluorescence dual labeling of TLR4 (green), Hsp70 (red), and colocalization (yellow) taken at 60× magnification, displayed with a scale of 10 μm in the liver sections from Chow, Chow + PP2A inhibitor, MASLD, MASLD + PP2A inhibitor, and NLRP3 KO MASLD + PP2A inhibitor mouse samples. Some of the colocalization events were indicated by white arrows. (**B**) Morphometry of Hsp70–TLR4 colocalization (yellow) events in all the groups were measured as arbitrary fluorescent units from three separate microscopic fields and plotted along the ordinate. All *p*-values were determined through one-way ANOVA with the significance level set at *p* < 0.05.

The events of Hsp70–TLR4 ligand binding in the liver were elucidated via immunofluorescence where TLR4 (green) and Hsp70 (red) were found to colocalize (yellow), and the number of events was measured in the liver sections of different experimental mice groups (Figure 4A). The highest reactivity in terms of Hsp70–TLR4 colocalization, indicative of the ligand binding events, was observed in the MASLD + PP2A inhibitor group, followed by the Chow + PP2A inhibitor group when compared to the MASLD and Chow groups (Figure 4A,B; *p* < 0.001). The absence of NLRP3 decreased the Hsp70 secretion; hence, the events of Hsp70–TLR4 colocalization decreased significantly in the NLRP3 KO MASLD + PP2A inhibitor group when compared to the MASLD + PP2A inhibitor group (Figure 3A,B and Figure 4A,B; *p* < 0.001). The absence of NLRP3 may also dictate lower Hsp70 protein levels, and thus drive lower TLR4 binding.

### 2.5. Hsp70–TLR4 Ligand Binding Regulated the NF-κB Phosphorylation to Manifest Hepatic Inflammation

**Figure 5:**
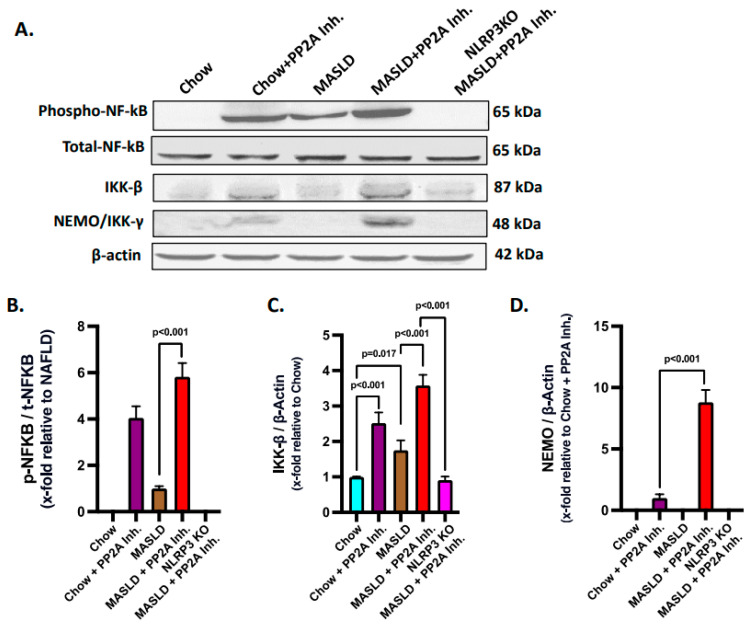
Hsp70–TLR4 ligand binding regulates the NF-κB phosphorylation to manifest hepatic inflammation. (**A**) Immunoblot analysis of phospho-NF-κB, total-NF-κB, IKK-β, NEMO/IKK-γ, and β-actin protein level expressions from the liver lysates of Chow, Chow + PP2A inhibitor, MASLD, MASLD + PP2A inhibitor, and NLRP3 KO MASLD + PP2A inhibitor mouse samples. (**B**) Densitometric analysis of phospho-NF-κB reactivity calculated as the ratio of phospho-NF-κB to total-NF-κB, displayed as mean and deviation (*n* = 3 in biological replicates). All *p*-values were determined through one-way ANOVA with the significance level set at *p* < 0.05. (**C**) Densitometric analysis of IKK-β reactivity calculated as the ratio of IKK-β to β-actin, displayed as mean and deviation (*n* = 3 in biological replicates). All *p*-values were determined through one-way ANOVA with the significance level set at *p* < 0.05. (**D**) Densitometric analysis of NEMO/IKK-γ reactivity calculated as the ratio of NEMO/IKK-γ to β-actin, displayed as mean and deviation (*n* = 3 in biological replicates). All *p*-values were determined through one-way ANOVA with the significance level set at *p* < 0.05.

TLR4 is well characterized in activating the NF-κB pathway, which involves phosphorylation and nuclear import of an active NF-κB complex to enhance the transcription of the genes related to immune response and inflammation [46,57]. In hepatocytes, the functional attributes of TLR4 have long been identified in the disease models of hepatic injury and MASH [47,48]. The Hsp70–TLR4 axis is known to activate NF-κB downstream [44]. In a previous study, extracellular Hsp 72 was shown to bind TLR2 and TLR4, but the precise role of a NLRP3–Hsp70–TLR4 axis has never been reported [58]. Hence, the NF-κB phosphorylation status, a downstream event of TLR4 activation, was probed via immunoblotting in the liver tissues of experimental mice groups. Results indicated that the phosphorylation of NF-κB (phospho-NF-κB, normalized against total-NF-κB) was highest in the MASLD + PP2A inhibitor group, followed by the Chow + PP2A inhibitor group, and almost absent in the NLRP3 KO MASLD + PP2A inhibitor group when compared with the MASLD + PP2A inhibitor group (Figure 5A,B; *p* < 0.001). Other components of the NF-κB pathway were also probed via the immunoblot technique, and it was observed that the IKK-β and NEMO/IKK-γ expression levels were correlated with the phospho-NF-κB profile with the highest expression in the MASLD + PP2A inhibitor group, followed by the Chow + PP2A inhibitor group, and decreased sharply in the NLRP3 KO MASLD + PP2A inhibitor group (Figure 5A,C,D; *p* < 0.001).

### 2.6. NLRP3–Hsp70–TLR4 Axis Triggered Cell Death and Survival Pathways in the Liver Following PP2A Inhibition by MC-LR

**Figure 6:**
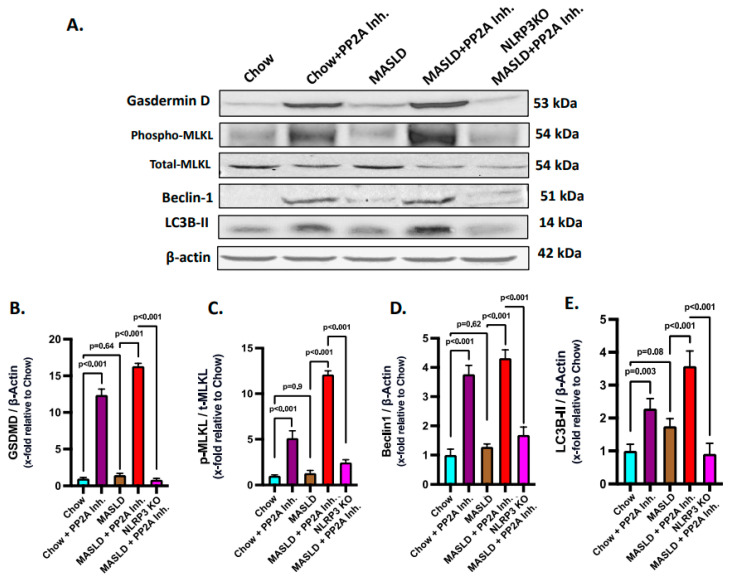
NLRP3–Hsp70–TLR4 axis shifts the fulcrum of cell death and survival pathways in the liver. (**A**) Immunoblot analysis of gasdermin D, phospho-MLKL, total-MLKL, Beclin 1, LC3B-II, and β-actin protein level expressions from the liver lysates of Chow, Chow + PP2A inhibitor, MASLD, MASLD + PP2A inhibitor, and NLRP3 KO MASLD + PP2A inhibitor mouse samples. (**B**) Densitometric analysis of gasdermin D reactivity calculated as the ratio of gasdermin D to β-actin, displayed as mean and deviation (*n* = 3 in biological replicates). All *p*-values were determined through one-way ANOVA with the significance level set at *p* < 0.05. (**C**) Densitometric analysis of phospho-MLKL reactivity calculated as the ratio of phospho-MLKL to total-MLKL, displayed as mean and deviation (*n* = 3 in biological replicates). All *p*-values were determined through one-way ANOVA with the significance level set at *p* < 0.05. (**D**) Densitometric analysis of Beclin 1 reactivity calculated as the ratio of Beclin 1 to β-actin, displayed as mean and deviation (*n* = 3 in biological replicates). All *p*-values were determined through one-way ANOVA with the significance level set at *p* < 0.05. (**E**) Densitometric analysis of LC3B-II reactivity calculated as the ratio of LC3B-II to β-actin, displayed as mean and deviation (*n* = 3 in biological replicates). All *p*-values were determined through one-way ANOVA with the significance level set at *p* < 0.05.

Gasdermin D plays an execution role in the pyroptotic cell death pathway in conjunction with NLRP3 and is required for IL-1β release [59]. Thus, NLRP3 inflammasome activation in turn synchronizes with pyroptosis and gasdermin D levels. Based on the above rationale, pyroptosis status was probed by immunoblotting and it was observed that the MASLD + PP2A inhibitor group showed the highest expression of gasdermin D, followed by the Chow + PP2A inhibitor group, and significantly decreased in the NLRP3 KO MASLD + PP2A inhibitor group (Figure 6A,B; *p* < 0.001).

Ascertaining the role of the NLRP3–TLR4–Hsp70 axis in the cell death mechanisms in MASLD to MASH progression was based on the reports that TLR4 activation is known to cause necroptosis via MLKL activation [60]. Phosphorylated MLKL is a key marker of necroptosis, which is a form of programmed cell death. Thus, an investigation was performed to check whether the NLRP3–Hsp70–TLR4 axis drives the necroptotic cell death in the liver with MASLD condition when PP2A is inhibited in the presence or absence of NLRP3 activation. Phosphorylation of the MLKL (phospho-MLKL, normalized against total-MLKL) was probed via immunoblot analysis from the liver lysates of the respective mice groups, and the highest level was observed that in the MASLD + PP2A inhibitor group, followed by the Chow + PP2A inhibitor group, whereas it was decreased significantly in the NLRP3 KO MASLD + PP2A inhibitor group (Figure 6A,C; *p* < 0.001).

Beclin 1 and LC3B-II are important proteins involved in the process of autophagy, which is a cellular mechanism responsible for the degradation and recycling of damaged or unnecessary cellular components [61]. The induction of autophagy in the liver tissues of experimental animal groups was measured in terms of elevated expression of the markers Beclin 1 and LC3B-II. The Beclin 1 and LC3B-II protein expression levels were probed via the immunoblot analyses and it was observed that both of their protein level expressions were highest in the MASLD + PP2A inhibitor group, followed by the Chow + PP2A inhibitor group, and decreased significantly in the NLRP3 KO MASLD + PP2A inhibitor group (Figure 6A,D,E; *p* < 0.001).

### 2.7. Hsp70–TLR4 Ligand Binding Augmented in Hepatocytes When Pre-Treated with Leptin

Our in vivo data mostly identified hepatocytes as the seat for NLRP3–Hsp70 co-binding as studied by immunofluorescence microscopy. Similarly, expression of Hsp70 and its binding to TLR4 appeared to be in the hepatocytes, though other cell participation could not be ruled out. To validate the crucial mechanism obtained from the in vivo results, the H2.35 mouse hepatocyte cell line was used to conduct the in vitro experiments. As we have shown before, the MASLD-like pathology is induced in the cells by treating with recombinant mouse leptin (100 ng/mL) for 24 h followed by recombinant mouse Hsp70 (50 μg/mL) treatment for an hour to stimulate the TLR4 signaling pathway [62]. The rationale for using leptin incubation rests purely on the basis of clinical leptin resistance in the liver microenvironment in MASLD. Cells were grouped in four sets of Control, Leptin (only leptin treated), Hsp70 (only Hsp70 treated), and Leptin + Hsp70 (Leptin treated followed by Hsp70 stimulation).

**Figure 7:**
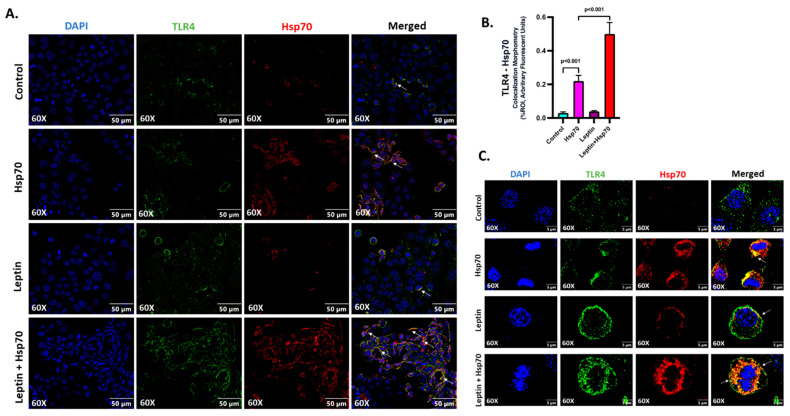
Hsp70–TLR4 ligand binding augments in H2.35 hepatocytes when pre-treated with leptin. (**A**) Immunofluorescence dual labeling of TLR4 (green), Hsp70 (red), and colocalization (yellow) taken at 60× (oil) magnification displayed with a scale of 50 μm in the H2.35 hepatocytes from Control, Hsp70, Leptin, and Leptin + Hsp70 groups. Some of the colocalization events are indicated by white arrows. (**B**) Morphometry of TLR4–Hsp70 colocalization (yellow) events in all the groups were measured as arbitrary fluorescent units from six separate microscopic fields and plotted along the ordinate. All *p*-values were determined through one-way ANOVA with the significance level set at *p* < 0.05. (**C**) Immunofluorescence dual labeling of TLR4 (green), Hsp70 (red), and colocalization (yellow) with DAPI (blue) counterstained taken at 60× (oil) magnification, displayed in zoom with a scale of 5 μm in the H2.35 hepatocytes in Hsp70 and Leptin + Hsp70 groups. Some of the colocalization events were indicated by white arrows.

Hsp70 (red) and TLR4 (green) ligand binding events were investigated on the cell surface, and the colocalization (yellow) profiles were generated to assess the Hsp70–TLR4 ligand binding (Figure 7A). A significant increase in the colocalization events was observed in the Leptin + Hsp70 group when compared to the Hsp70-only group (Figure 7A,B; *p* < 0.001). The higher magnification images displayed separately also show the prominence of the Hsp70–TLR4 interaction, indicative of ligand binding in discrete colocalization profiles (Figure 7C).

### 2.8. Leptin Elevated the Expression of TLR4 to Increase the Hsp70–TLR4 Ligand Binding and Subsequent NF-κB Phosphorylation in Hepatocytes

**Figure 8:**
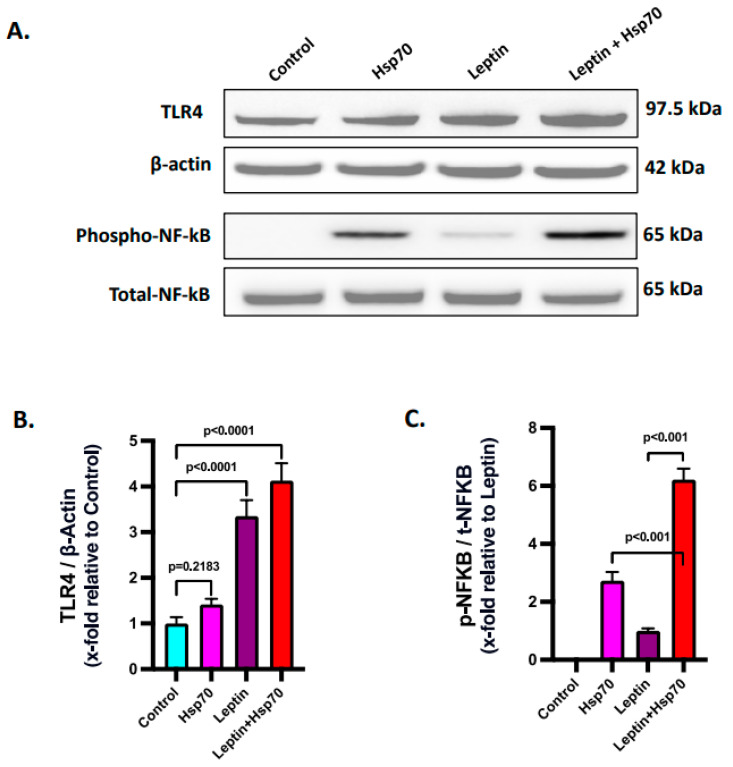
Leptin elevates the expression of TLR4 to increase the Hsp70–TLR4 ligand binding and subsequent NF-κB phosphorylation. (**A**) Immunoblot analysis of TLR4, phospho-NF-κB, total-NF-κB, and β-actin protein level expressions in the H2.35 hepatocytes from Control, Hsp70, Leptin, and Leptin + Hsp70 groups. (**B**) Densitometric analysis of TLR4 reactivity calculated as the ratio of TLR4 to β-actin, displayed as mean and deviation (*n* = 3 in biological replicates). All *p*-values were determined through two-way ANOVA with the significance level set at *p* < 0.05. (**C**) Densitometric analysis of phospho-NF-κB reactivity calculated as the ratio of phospho-NF-κB to total-NF-κB, displayed as mean and deviation (*n* = 3 in biological replicates). All *p*-values were determined through one-way ANOVA with the significance level set at *p* < 0.05.

Leptin was reported to increase the expression of TLR4 [63]. Hence, investigations were made to check if leptin caused increased TLR4 expression and a resulting Hsp70–TLR4 ligand binding in the H2.35 mouse hepatocytes. Immunoblot analysis revealed a significantly increased protein level expression of TLR4 was observed with 24 h leptin treatment in the Leptin and Leptin + Hsp70 groups when compared to the Control and Hsp70-only groups (Figure 8A,B; *p* < 0.001). Consequently, NF-κB phosphorylation was also found to be increased in the Leptin + Hsp70 group compared to the Hsp70-only group (Figure 8A,C; *p* < 0.001). Thus, the results elucidated how NF-κB phosphorylation and subsequent inflammatory events are dependent on the elevated leptin-dependent TLR4 expression and subsequent increase in Hsp70–TLR4 ligand binding in H2.35 mouse hepatocytes.

## 3. Discussion

This study has provided for the first time a mechanistic insight into the NLRP3–Hsp70–TLR4 axis in MASLD and its progression to MASH upon PP2A inhibition by environmental toxicant exposure. NLRP3 inflammasome activation induced Hsp70 production and release, which then interacted with TLR4 on hepatocytes, resulting in the activation of NF-κB and promoting inflammation. In the absence of NLRP3, these effects were attenuated. Increased fat accumulation and leptin resistance in MASLD upregulated TLR4 expression, while PP2A inhibition enhanced Hsp70 production, contributing to heightened Hsp70–TLR4 binding and NF-κB activation. In vitro results further confirmed this mechanism, showing increased inflammation in Hsp70-stimulated hepatocytes with leptin pre-treatment.

The existing scientific premise established by our lab demonstrated that the pathology of MASLD is mainly associated with oxidative stress and inflammation in exerting liver damage and the release of inflammatory messengers at a systemic level which further expand to ectopic injury including gut, kidney, and brain [64,65,66,67]. On the other hand, recent cyanobacterial blooms have raised concerns as they release protein phosphatase 2A (PP2A) inhibitors like MC-LR, posing a significant threat to individuals with MASLD [18]. Past studies reported by our group and others have thoroughly demonstrated the impacts of PP2A inhibitors and their byproducts in exacerbating the pathophysiology of MASLD and its progression toward MASH as referenced earlier. Conclusively, MC-LR produced by cyanobacteria aggravates the amplitude of this hepatic injury. One of the reasons for the exacerbating pathology was identified as inflammation. Here, we exclusively elucidated that in the underlying conditions of MASLD, the second hit of PP2A inhibition causes amplified inflammation, cell death, and fibrotic damage to the liver via NLRP3 inflammasome activation that triggers an Hsp70-induced TLR4 activation.

Previous studies from our laboratory demonstrated that activation of the NLRP3 inflammasome induces inflammation in the liver and brain tissues of MASLD/MASH mouse models. NLRP3 inflammasome activation is associated with immune cell activation, hepatocyte damage, and hepatic inflammation in various acute and chronic liver diseases [68,69,70,71]. Furthermore, our past research revealed that PP2A inhibitors trigger NLRP3 activation in intestinal and liver tissues [5,7,29,72]. To date, there is significant evidence linking heat shock proteins (HSPs) to inflammatory events and immune regulation [30,73]. HSPs have been found to modulate various inflammasomes, including NLRP3 [41]. When released extracellularly, Hsp70 acts as a DAMP and triggers inflammation whereas intracellular Hsp70’s anti-inflammatory functions are compromised in MASLD [42,49]. PP2A inhibitors are known to increase Hsp70 expression [50]. However, the precise involvement and characterization of Hsp70 in MASLD and PP2A inhibition remain unclear. Therefore, we investigated the mechanistic relationship between NLRP3 and Hsp70 by examining the contribution of extracellular Hsp70 to pathophysiology in the absence of NLRP3 (NLRP3KO). In our current research we demonstrated a detailed mechanism involving the NLRP3 inflammasome, and how it pertains to the unique contraption of Hsp70 production and release has been substantiated with the TLR4 pathway and subsequent activation of pro-inflammatory NF-κB pathway in manifesting the disease pathology. Thus, we probed further and observed that NLRP3 drives and determines the production of Hsp70 in the liver and its systemic level release. The presence of NLRP3 avails Hsp70 to bind with the TLR4 receptors, expressed mainly by the hepatocytes in the liver, which further causes downstream phosphorylation of NF-κB and activates the proinflammatory cascade, contributing to the pathophysiology of MASLD and instigates its worsening towards steatohepatitis. Lack of NLRP3 in the system markedly decreases the rate of Hsp70 production in the liver and its circulatory concentration which renders its availability to the hepatocytes for Hsp70–TLR4 ligand binding and subsequent NF-κB activation.

NLRP3 is the upstream regulator of Hsp70 production and release [52], known to modulate the proinflammatory fate caused by Hsp70. This inflammatory insult is known to impact cell survival and confers more damage by inducing cell death; hence, we identified the NLRP3–Hsp70–TLR4 axis to play a pivotal role in determining the stages of liver injury and fibrosis. MASLD conditions cause increased accumulation of fat and leptin resistance in the liver, which amplifies the protein level of expression of TLR4. The PP2A inhibitor increases the production of Hsp70 in the liver and its release at the systemic level, thus enhancing the availability of Hsp70 in liver tissue and finally to the hepatocytes, which consequently serves to ensure maximal Hsp70–TLR4 ligand binding and resultant NF-κB activation. Our in vitro results indicated this mechanism underlying the immoderate inflammation in Hsp70-stimulated hepatocytes when pre-treated with leptin. Hsp70 has been elucidated as a TLR4 ligand and instigates proinflammatory response [44,45] and our results indicated the same: lack of secretory Hsp70 and its reduced binding to TLR4 plays the pivotal role in activating inflammatory cascades in the liver. This also justified the elevation of underlying inflammation in MASLD to MASH progression when PP2A is inhibited by cyanotoxins.

Cell death and survival decisions are crucially dependent on the amplitude of tissue injury and inflammation. Important aspects of that are covered in this study, including fundamental pathways of pyroptosis, necroptosis, and autophagy. PP2A inhibitors are reported to cause pyroptosis via NLRP3 activation in hepatocytes [24]. In our results, it has been reflected that the lack of NLRP3 activation in the NLRP3 KO MASLD + PP2A Inh. group affected the pyroptosis pathway and associated cell death. Consequently, hepatic cell death from pyroptosis is regulated in the absence of NLRP3 but is recorded to be highest in the MASLD + PP2A Inh. group. Necroptotic cell death is a hallmark of cell and tissue level injury due to augmented inflammation and PP2A inhibitor has also been identified prior in causing necroptosis in mouse primary hepatocytes [74]. Phosphorylated MLKL is known to execute NLRP3 inflammasome activation in a cell-intrinsic manner [75]. Both Hsp70 and TLR4 activation are elucidated in the activation of MLKL and executing necroptotic cell death [76]. We also observed the highest level of necroptosis in the MASLD + PP2A Inh. group and it decreased markedly in the NLRP3 KO MASLD + PP2A Inh. group. Hence, we could explain here that, due to the NLRP3 knockout, the levels of Hsp70 and TLR4 activation by its ligand binding are significantly less, preventing immoderate inflammation and subsequent cell death by necroptosis. The MASLD condition and PP2A inhibitors are known to induce autophagy to combat ER stress, cellular damage, and inflammation, both in vivo and in vitro [61,77]. Autophagy can regulate the NLRP3 activation and ameliorate the inflammatory damage that occurs via NLRP3 [78]. Also, NLRP3 inflammasome activation and autophagy have been correlated in hepatic stellate cell activation and liver fibrosis [79]. Hence, in accordance with the existing research, our results also demonstrated that the status of autophagy in experimental mice liver samples was dependent on the level of tissue injury, inflammation, and cell death mediated through the NLRP3–Hsp70–TLR4 axis.

Though our study provides important insight into the NLRP3–Hsp70–TLR4 axis in determining the poor outcome in MASLD, we are limited in extrapolating the role of Hsp70 in general. As is known, Hsp70 has been shown to act as an anti-inflammatory molecule, albeit intracellularly. It might be the case that the intracellular Hsp70 expression and/or release in the extracellular matrix may be tightly regulated. Interestingly, obesity shows a suppressed anti-inflammatory activity of Hsp70 [49], and our present research did not study the exact source of Hsp70, although we showed that both the liver expression and serum levels of Hsp70 were under the tight regulation of NLRP3. It might be justified to assume that there are other molecular mediators of Hsp70 that modulate its pro- or anti-inflammatory actions that may have broader implications in liver disease pathology. Based on our initial findings of higher serum level Hsp70 that was tightly regulated by NLRP3, we focused more on the secretory Hsp70, which is pro-inflammatory in nature [42,43]. A detailed mechanism of NLRP3 in regulating Hsp70 production and release into the liver microenvironment is another major domain of interest that we would like to study in the future. Additionally, for future studies, it would be appropriate to conduct advanced validation methods including transient and stable Hsp70 knockdown in vitro models using small interfering RNAs (siRNA) and short hairpin RNAs (shRNA), respectively, targeting the inducible Hsp70 isoforms. Being an important ubiquitous chaperone protein, the generation of a Hsp70 knockout system can be detrimental for cell survival; hence, it may be apt to use efficient knockdown systems only. To further establish the role of Hsp70 in driving the NLRP3–Hsp70–TLR4 axis, it will be important to use a transcriptomics approach to better understand global gene expression changes resulting from the knockdown or inhibition of Hsp70 in the context of MASLD and PP2A inhibition.

In summary, our proposed mechanism for liver injury, involving the NLRP3–Hsp70–TLR4 axis, has significant implications for the development and repurposing of therapeutics in the treatment of hepatic inflammation in patients with MASLD and progressive steatohepatitis. Animal studies have demonstrated that NLRP3 inhibitors known to be effective in clinical settings can halt the progression of liver damage and fibrosis in MASLD/MASH models [80,81,82]. Additionally, previous research has shown that TLR4 null mice exhibit reduced injury and lipid accumulation in models of steatohepatitis [83]. Our findings further underscore the crucial role of TLR4 in driving the transition from MASLD to MASH. The stimulation of TLR4 by secretory Hsp70 initiates proinflammatory reactions and increases the burden of inflammation. Thus, targeting the NLRP3–Hsp70–TLR4 axis with specific clinically developed antagonists offers a focused approach in the clinical management of MASLD and MASH, though the use of Hsp70 inhibitors may not be a correct approach owing to its dual roles in pathology.

## 4. Materials and Methods

The MC-LR (henceforth, also referred to as PP2A inhibitor) was acquired from Cayman Chemical Company (Ann Arbor, MI, USA). Primary antibodies against Cluster of Differentiation 68 (CD68), α-smooth muscle actin (α-SMA), Hsp70, and secondary antibodies conjugated with horseradish peroxidase (HRP) were obtained from Abcam (Cambridge, MA, USA). Primary antibodies against NLRP3, ASC2, IL-1β, TLR4, IKK-β, NEMO/IKK-γ, and β-actin were purchased from Santacruz Biotechnology (Dallas, TX, USA). Primary antibodies against phospho-NF-κB, total-NF-κB, phospho-mixed lineage kinase domain-like protein (MLKL), total-MLKL, Beclin 1, LC3B-II, and gasdermin D were obtained from Cell Signaling Technology (Beverly, MA, USA). Species-specific biotinylated secondary antibodies and streptavidin-HRP were acquired from Vector Laboratories (Vectastain Elite ABC kit, Newark, CA, USA). Fluorescence-conjugated (Alexa Flour) secondary antibodies and ProLong Gold antifade mounting media with DAPI were purchased from Thermo Fisher Scientific (Grand Island, NY, USA). The IHC-Tek was obtained from IHCWORLD (Catalog# IW-1000). The liver tissue sections were paraffin-embedded by AML Laboratories (St. Augustine, FL, USA). Recombinant mouse Hsp70 was purchased from Enzo Life Sciences (Farmingdale, NY, USA). Unless otherwise specified, all other chemicals used in this study were purchased from Sigma-Aldrich (St. Louis, MO, USA).

### 4.1. Animal Model

This study used pathogen-free, adult, male, 8 weeks old C57BL/6J wild-type (WT) mice and NLRP3 knockout (NLRP3 KO) mice obtained from the Jackson Laboratories (Bar Harbor, ME, USA). The mice were used to investigate the effects of MASLD induction. The WT and NLRP3 KO mice (B6.129S6-*Nlrp3*^tm1Bhk^/J, Male, 8 weeks, Genetic Background: C57BL/6J) were fed a high-fat diet (HFD) containing 60% kCal for 6 weeks until the age of 14 weeks in mice to induce MASLD. A separate group of mice was fed a normal chow diet, which served as the control group for the study. The mice were housed in a temperature-controlled room with a 12 h light/dark cycle and had ad libitum access to food and water. All experimental procedures were conducted following the guidelines outlined in the NIH Guide for the Humane Care and Use of Laboratory Animals. After the completion of the experimental treatments, all mice were euthanized.

### 4.2. Experimental Models Used

This study involved the following experimental groups:

i. Chow: WT mice fed a chow diet only.
ii. Chow + PP2A Inh.: WT mice fed a chow diet and exposed to PP2A inhibitor.
iii. MASLD: WT mice fed an HFD (60% kCal) obtained from Research Diets (New Brunswick, NJ, USA) to induce MASLD.
iv. MASLD + PP2A Inh.: WT mice fed a high-fat diet and exposed to PP2A inhibitor.
v. NLRP3 KO MASLD + PP2A Inh.: NLRP3 knockout mice exposed to PP2A inhibitor and fed an HFD.

At 14 weeks of age, mice from the Chow + PP2A Inh., MASLD + PP2A Inh., and NLRP3 KO MASLD + PP2A Inh. groups were administered with PP2A inhibitor via oral gavage at a dose of 10 μg/kg body weight. The administration of the PP2A inhibitor was continued for 2 weeks, with a frequency of 5 doses per week. The above dose was selected based on the NOAEL established (40 μg/kg/day) in mice [84]. Also, several laboratories including ours have used a dose ranging between 10–120 μg/kg body weight as a subchronic dose for hepatotoxicity [53]. Early mortality was observed in both the 50 μg/kg (1/17, 6%), and 100 μg/kg (3/17, 18%) MC-LR exposed mice in a study that used a leptin receptor knockout model of MASLD [54], and most importantly, dissolved MCs were found in all the water samples taken in Meiliang Bay of Lake Taihu that ranged from 3.22 to 11.47 μg/L [55]. Based on the above blood levels in humans, the same study used MC-LR concentrations of 5 μg/Kg and 20 micrograms/kg -body weight as a subchronic dose mimicking a human exposure [55]. The PP2A inhibitor was dissolved in a vehicle consisting of ethanol and PBS. The total number of animals in each group (*n* = 6) was determined based on statistical power calculations to ensure adequate statistical analysis. The mice were randomly allocated to their respective cages following a randomization procedure. At 16 weeks of age, all mice were euthanized, and serum and liver tissues were collected for further analysis. Liver tissues were fixed in 10% neutral buffered formalin after euthanization to prepare them for sectioning and subsequent processing.

### 4.3. Cell Culture

The H2.35 mouse hepatocyte cell line (Cat# 94050407-1VL) was acquired from Sigma-Aldrich (St. Louis, MO, USA). The cells were cultured in Dulbecco’s Modified Eagle’s Medium (DMEM) obtained from Corning (Tewksbury, MA, USA). The DMEM medium was supplemented with 100 U/mL of penicillin, 100 μg/mL of streptomycin from Gibco (Grand Island, NY, USA), 5% fetal bovine serum (FBS), 2 mM glutamine, and 250 nM dexamethasone (DXMT). The cells were maintained at a temperature of 39 °C in a humidified atmosphere with 5% CO_2_. Before initiating the treatment, the cells underwent serum starvation overnight using DMEM containing 0.25% FBS. The control group of cells was treated with vehicle control. Leptin and Leptin + Hsp70 group of cells were pre-treated with mouse leptin at a concentration of 100 ng/mL for 24 h. Cells were then stimulated with recombinant mouse Hsp70 for 1 h at a concentration of 50 μM with or without leptin pre-treatment. Once all the treatments were completed, the cells were subjected to immunoblotting and immunofluorescence, and cell supernatants were collected and stored at −80 °C for future use.

### 4.4. Immunohistochemistry

After deparaffinizing the formalin-fixed, paraffin-embedded liver tissue sections using a standard laboratory protocol, the antigen epitope retrieval was carried out using an epitope retrieval solution and a steamer from IHC World (Woodstock, MD, USA). To block the endogenous peroxidase activity, a 3% H_2_O_2_ solution was applied for 20 min. Subsequently, serum blocking was performed using 5% goat serum for 1 h. The tissue sections were then incubated overnight at 4 °C with primary antibodies for CD68, α-SMA, and IL-1β, diluted according to the recommended dilutions in a blocking buffer. This incubation was carried out in a humidified chamber. For detection, species-specific biotinylated secondary antibodies and streptavidin conjugated with horseradish peroxidase were used, following the manufacturer’s standard protocols. To visualize the immunoreactivity, 3,3-diaminobenzidine (DAB) from Sigma-Aldrich was used as a chromogenic substrate. The sections were counterstained with Mayer’s hematoxylin from Sigma-Aldrich (St. Louis, MO, USA). Throughout the procedure, the tissue sections were washed with 1× PBS-T (PBS + 0.05% Tween 20) between the steps to remove any unbound reagents. Finally, the sections were mounted in Aqua Mount from Lerner Laboratories (Kalamazoo, MI, USA). Images were acquired using an Olympus BX63 microscope (Olympus, Center Valley, PA, USA). Morphometry analysis was performed using cellSens software V2.2 (Olympus, Center Valley, PA, USA).

### 4.5. Immunofluorescence

In the in vivo experiments, formalin-fixed, paraffin-embedded liver tissue sections were deparaffinized following standard instructions. Epitope retrieval of the deparaffinized tissue sections was performed using an epitope retrieval solution and a steamer from IHC World, following the manufacturer’s protocol. Primary antibodies for NLRP3, ASC2, Hsp70, and TLR4 were used at recommended dilutions and incubated overnight at 4 °C. Species-specific anti-IgG secondary antibodies conjugated with Alexa Fluor 633 or 488 from Invitrogen (Carlsbad, CA, USA) were used for detection. The tissue sections were mounted using ProLong Gold antifade mounting media with DAPI from Thermo Fisher Scientific (Grand Island, NY, USA) which helps preserve fluorescence and provides nuclear staining with DAPI. Images of the tissue sections were captured under 40× magnification using an Olympus BX63 microscope (Olympus, Center Valley, PA, USA). Morphometry analysis was performed using cellSens software V2.2 (Olympus, Center Valley, PA, USA).

For the in vitro experiments, upon completion of the treatments, the cells attached to coverslips were fixed with a pre-warmed 4% paraformaldehyde solution for 10 min at room temperature. The cells were then permeabilized with PBS containing 0.1% Triton X and blocked with a solution containing 3% BSA, 0.2% Tween, and 10% FBS in PBS. The cells were incubated with primary antibodies against TLR4 and Hsp70 at recommended dilutions, washed thoroughly, and incubated with species-specific Alexa Fluor 633 and 488 secondary antibodies (diluted at 1:150). The stained cells attached to the coverslips were mounted on slides using ProLong Gold antifade mounting media with DAPI from Thermo Fisher Scientific (Grand Island, NY, USA). Images of the stained cells were captured under 60× magnification (oil immersion) using an Olympus BX63 microscope from Olympus (Center Valley, PA, USA). Morphometry analysis was performed using cellSens software V2.2 from Olympus (Center Valley, PA, USA).

### 4.6. Quantitative Real-Time Polymerase Chain Reaction

To measure gene expression levels in liver tissue samples, the qRT–PCR protocol was followed. Initially, the liver tissue was homogenized and centrifuged to eliminate any extraneous tissue particles. Total RNA was then extracted from the homogenized liver tissue using TRIzol reagent (Invitrogen) according to the manufacturer’s instructions. The extracted RNA was subsequently purified using RNAse mini kit columns from Qiagen (Valencia, CA, USA). Following purification, 1000 ng of the purified RNA was reverse transcribed into cDNA using the iScript cDNA synthesis kit (Bio-Rad, Hercules, CA, USA). For the qRT–PCR analysis, gene-specific primers and SsoAdvanced SYBR Green Supermix (Bio-Rad) were utilized in a CFX96 thermal cycler (Bio-Rad, Hercules, CA, USA). The amplification was carried out through a series of thermal cycles optimized for specific primers. The resulting fluorescence signals were monitored, and the threshold cycle (C_t_) values were determined. To normalize the data, the target genes’ C_t_ values were compared to those of the internal control gene, GAPDH. The relative fold change in gene expression was calculated using the 2^(−ΔΔCt)^ method to compare different experimental conditions or treatment groups. Primer sequences for the qRT–PCR analysis of the target genes are provided in Table 1.

### 4.7. Western Blot

Proteins from liver tissue samples and cell culture harvests were extracted using RIPA lysis buffer supplemented with 1× protease inhibitor and phosphatase inhibitor cocktail. The protein concentration was determined using the BCA kit (Thermo Fisher Scientific, Rockford, IL, USA). Approximately 30 μg of denatured protein was loaded onto each well of a Novex 4–12% bis-tris gradient gel and subjected to standard SDS-PAGE. The proteins were separated based on their molecular weight using SDS-PAGE. The resolved protein bands were then transferred from the gel to a nitrocellulose membrane using pre-cut nitrocellulose/filter paper sandwiches from Bio-Rad Laboratories (Hercules, CA, USA) and the Trans-Blot Turbo transfer system (Bio-Rad, Hercules, CA, USA). Afterward, the membrane was blocked with 5% bovine serum albumin (BSA) for 1 h to prevent nonspecific binding. Subsequently, the membrane was incubated overnight at 4 °C with primary antibodies targeting Hsp70, phospho-NF-κB, total-NF-κB, phospho-MLKL, total-MLKL, Beclin 1, LC3B-II, gasdermin D, TLR4, and β-actin at the recommended dilutions. Following primary antibody incubation, the membrane was incubated with species-specific horseradish peroxidase-conjugated secondary antibodies (diluted 1:5000) for 1.5 h. For protein band visualization, the Pierce ECL Western Blotting substrate (Thermo Fisher Scientific, Rockford, IL, USA) was used. The blot images were captured using the G: Box Chemi XX6 and Biorad ChemiDoc MP imaging system, and densitometry analysis was performed using Image J software V1.53t. This analysis allowed quantification of the protein bands and assessment of relative protein expression levels.

### 4.8. ELISA

Serum levels of Hsp70 were estimated in the serum samples collected from the mice groups using a commercially available mouse heat shock protein 70 (HSPA4) ELISA kit (cat# RK06376) from Abclonal (Woburn, MA, USA).

### 4.9. Statistical Analyses

The results are presented as mean ± standard error of the mean (S.E.M.). Statistical analyses were performed using one-way analysis of variance (ANOVA) and unpaired *t*-tests, and significance was determined by the Bonferroni–Dunn post hoc correction method using GraphPad Prism V10.1.0 Software, Inc. (San Diego, CA, USA). A *p*-value of less than 0.05 (*p* < 0.05) was considered statistically significant.

## Figures and Tables

**Table 1:** qRT-PCR Primer sequences for target genes.

Genes	Forward Primer Sequence	Reverse Primer Sequence	Source
*Hsp70*	CAGCGAGGCTGACAAGAAGAA	GGAGATGACCTCCTGGCACT	[56]
*Hsp90*	TTGGTTACTTCCCCGTGCTG	GCCTTTTGCCGTAGGGTTTC	designed by us
*Hsp60*	CATCGGAAGCCATTGGTCATAA	CGTGCTTAGAGCTTCTCCGTCA	[85]
*Hsp25*	ACAGTGAAGACCAAGGAAGG	CTGGAGGGAGCGTGTATTT	designed by us
*HspH1*	GACCCTCAAGGAGTTCCATATC	CTCTCGACTTCTCTCCATCTTTC	designed by us
*HspB8*	AGACCCCTTTCGGGACTCA	GGCTGTCAAGTCGTCTGGAA	[86]
*GAPDH*	CGACTTCAACAGCAACTCCCACTCTTCC	TGGGTGGTCCAGGGTTTCTTACTCCTT	[87]

## Data Availability

Data are contained within the article and Supplementary materials.

## Supplementary Materials

The following supporting information can be downloaded at: https://www.mdpi.com/article/10.3390/ijms242216354/s1.

## References

[1] Bellentani S., Scaglioni F., Marino M., Bedogni G. (2010). Epidemiology of non-alcoholic fatty liver disease. Dig Dis..

[2] Mitra S., De A., Chowdhury A. (2020). Epidemiology of non-alcoholic and alcoholic fatty liver diseases. Transl. Gastroenterol. Hepatol..

[3] Wong V.W., Ekstedt M., Wong G.L., Hagstrom H. (2023). Changing epidemiology, global trends and implications for outcomes of NAFLD. J. Hepatol..

[4] Arciello M., Gori M., Maggio R., Barbaro B., Tarocchi M., Galli A., Balsano C. (2013). Environmental pollution: A tangible risk for NAFLD pathogenesis. Int. J. Mol. Sci..

[5] Albadrani M., Seth R.K., Sarkar S., Kimono D., Mondal A., Bose D., Porter D.E., Scott G.I., Brooks B., Raychoudhury S. (2019). Exogenous PP2A inhibitor exacerbates the progression of nonalcoholic fatty liver disease via NOX2-dependent activation of miR21. Am. J. Physiol. Gastrointest. Liver Physiol..

[6] Seth R.K., Kumar A., Das S., Kadiiska M.B., Michelotti G., Diehl A.M., Chatterjee S. (2013). Environmental toxin-linked nonalcoholic steatohepatitis and hepatic metabolic reprogramming in obese mice. Toxicol. Sci..

[7] Al-Badrani M., Saha P., Mondal A., Seth R.K., Sarkar S., Kimono D., Bose D., Porter D.E., Scott G.I., Brooks B. (2020). Early microcystin-LR exposure-linked inflammasome activation in mice causes development of fatty liver disease and insulin resistance. Environ. Toxicol. Pharmacol..

[8] Nagata K., Suzuki H., Sakaguchi S. (2007). Common pathogenic mechanism in development progression of liver injury caused by non-alcoholic or alcoholic steatohepatitis. J. Toxicol. Sci..

[9] Woolbright B.L., Williams C.D., Ni H., Kumer S.C., Schmitt T., Kane B., Jaeschke H. (2017). Microcystin-LR induced liver injury in mice and in primary human hepatocytes is caused by oncotic necrosis. Toxicon.

[10] Amado L.L., Monserrat J.M. (2010). Oxidative stress generation by microcystins in aquatic animals: Why and how. Environ. Int..

[11] Dawson R.M. (1998). The toxicology of microcystins. Toxicon.

[12] Huang H., Liu C., Fu X., Zhang S., Xin Y., Li Y., Xue L., Cheng X., Zhang H. (2016). Microcystin-LR Induced Apoptosis in Rat Sertoli Cells via the Mitochondrial Caspase-Dependent Pathway: Role of Reactive Oxygen Species. Front. Physiol..

[13] Agostini L., Martinon F., Burns K., McDermott M.F., Hawkins P.N., Tschopp J. (2004). NALP3 forms an IL-1beta-processing inflammasome with increased activity in Muckle-Wells autoinflammatory disorder. Immunity.

[14] Ogura Y., Sutterwala F.S., Flavell R.A. (2006). The inflammasome: First line of the immune response to cell stress. Cell.

[15] Tilg H., Moschen A.R. (2010). Evolution of inflammation in nonalcoholic fatty liver disease: The multiple parallel hits hypothesis. Hepatology.

[16] Zheng C., Zeng H., Lin H., Wang J., Feng X., Qiu Z., Chen J.A., Luo J., Luo Y., Huang Y. (2017). Serum microcystin levels positively linked with risk of hepatocellular carcinoma: A case-control study in southwest China. Hepatology.

[17] Zhao Y., Yan Y., Xie L., Wang L., He Y., Wan X., Xue Q. (2020). Long-term environmental exposure to microcystins increases the risk of nonalcoholic fatty liver disease in humans: A combined fisher-based investigation and murine model study. Environ. Int..

[18] Sauve S., Desrosiers M. (2014). A review of what is an emerging contaminant. Chem. Cent. J..

[19] MacKintosh C., Beattie K.A., Klumpp S., Cohen P., Codd G.A. (1990). Cyanobacterial microcystin-LR is a potent and specific inhibitor of protein phosphatases 1 and 2A from both mammals and higher plants. FEBS Lett..

[20] Honkanen R.E., Zwiller J., Moore R.E., Daily S.L., Khatra B.S., Dukelow M., Boynton A.L. (1990). Characterization of microcystin-LR, a potent inhibitor of type 1 and type 2A protein phosphatases. J. Biol. Chem..

[21] Xu Y., Xing Y., Chen Y., Chao Y., Lin Z., Fan E., Yu J.W., Strack S., Jeffrey P.D., Shi Y. (2006). Structure of the protein phosphatase 2A holoenzyme. Cell.

[22] Reynhout S., Janssens V. (2019). Physiologic functions of PP2A: Lessons from genetically modified mice. Biochim. Biophys. Acta Mol. Cell Res..

[23] Xian L., Hou S., Huang Z., Tang A., Shi P., Wang Q., Song A., Jiang S., Lin Z., Guo S. (2015). Liver-specific deletion of Ppp2calpha enhances glucose metabolism and insulin sensitivity. Aging.

[24] Zhang Y., Zhu P., Wu X., Yuan T., Su Z., Chen S., Zhou Y., Tao W.A. (2021). Microcystin-LR Induces NLRP3 Inflammasome Activation via FOXO1 Phosphorylation, Resulting in Interleukin-1beta Secretion and Pyroptosis in Hepatocytes. Toxicol. Sci..

[25] Maitiabula G., Tian F., Wang P., Zhang L., Gao X., Wan S., Sun H., Yang J., Zhang Y., Gao T. (2022). Liver PP2A-Calpha Protects From Parenteral Nutrition-associated Hepatic Steatosis. Cell Mol. Gastroenterol. Hepatol..

[26] Wu Y., Song P., Xu J., Zhang M., Zou M.H. (2019). Withdrawal: Activation of protein phosphatase 2A by palmitate inhibits AMP-activated protein kinase. J. Biol. Chem..

[27] Liangpunsakul S., Sozio M.S., Shin E., Zhao Z., Xu Y., Ross R.A., Zeng Y., Crabb D.W. (2010). Inhibitory effect of ethanol on AMPK phosphorylation is mediated in part through elevated ceramide levels. Am. J. Physiol. Gastrointest. Liver Physiol..

[28] Chen X.Y., Cai C.Z., Yu M.L., Feng Z.M., Zhang Y.W., Liu P.H., Zeng H., Yu C.H. (2019). LB100 ameliorates nonalcoholic fatty liver disease via the AMPK/Sirt1 pathway. World J. Gastroenterol..

[29] Sarkar S., Kimono D., Albadrani M., Seth R.K., Busbee P., Alghetaa H., Porter D.E., Scott G.I., Brooks B., Nagarkatti M. (2019). Environmental microcystin targets the microbiome and increases the risk of intestinal inflammatory pathology via NOX2 in underlying murine model of Nonalcoholic Fatty Liver Disease. Sci. Rep..

[30] Swanson K.V., Deng M., Ting J.P. (2019). The NLRP3 inflammasome: Molecular activation and regulation to therapeutics. Nat. Rev. Immunol..

[31] Spierings J., van Eden W. (2017). Heat shock proteins and their immunomodulatory role in inflammatory arthritis. Rheumatology.

[32] Moseley P.L. (1998). Heat shock proteins and the inflammatory response. Ann. N. Y. Acad. Sci..

[33] Martine P., Rebe C. (2019). Heat Shock Proteins and Inflammasomes. Int. J. Mol. Sci..

[34] Zhang J., Fan N., Peng Y. (2018). Heat shock protein 70 promotes lipogenesis in HepG2 cells. Lipids Health Dis..

[35] Rehati A., Abuduaini B., Liang Z., Chen D., He F. (2023). Identification of heat shock protein family A member 5 (HSPA5) targets involved in nonalcoholic fatty liver disease. Genes Immun..

[36] Sun Y., Meng G.M., Guo Z.L., Xu L.H. (2011). Regulation of heat shock protein 27 phosphorylation during microcystin-LR-induced cytoskeletal reorganization in a human liver cell line. Toxicol. Lett..

[37] Udono H., Srivastava P.K. (1993). Heat shock protein 70-associated peptides elicit specific cancer immunity. J. Exp. Med..

[38] Udono H., Srivastava P.K. (1994). Comparison of tumor-specific immunogenicities of stress-induced proteins gp96, hsp90, and hsp70. J. Immunol..

[39] Cosemans G., Merckx C., De Bleecker J.L., De Paepe B. (2022). Inducible Heat Shock Protein 70 Levels in Patients and the mdx Mouse Affirm Regulation during Skeletal Muscle Regeneration in Muscular Dystrophy. Front. Biosci. Schol. Ed..

[40] Boudesco C., Cause S., Jego G., Garrido C. (2018). Hsp70: A Cancer Target Inside and Outside the Cell. Methods Mol. Biol..

[41] Martine P., Chevriaux A., Derangere V., Apetoh L., Garrido C., Ghiringhelli F., Rebe C. (2019). HSP70 is a negative regulator of NLRP3 inflammasome activation. Cell Death Dis..

[42] Hulina A., Grdic Rajkovic M., Jaksic Despot D., Jelic D., Dojder A., Cepelak I., Rumora L. (2018). Extracellular Hsp70 induces inflammation and modulates LPS/LTA-stimulated inflammatory response in THP-1 cells. Cell Stress Chaperones.

[43] Ren B., Zou G., Huang Y., Xu G., Xu F., He J., Zhu H., Yu P. (2016). Serum levels of HSP70 and other DAMP proteins can aid in patient diagnosis after traumatic injury. Cell Stress Chaperones.

[44] Fang H., Wu Y., Huang X., Wang W., Ang B., Cao X., Wan T. (2011). Toll-like receptor 4 (TLR4) is essential for Hsp70-like protein 1 (HSP70L1) to activate dendritic cells and induce Th1 response. J. Biol. Chem..

[45] Theivanthiran B., Yarla N., Haykal T., Nguyen Y.V., Cao L., Ferreira M., Holtzhausen A., Al-Rohil R., Salama A.K.S., Beasley G.M. (2022). Tumor-intrinsic NLRP3-HSP70-TLR4 axis drives premetastatic niche development and hyperprogression during anti-PD-1 immunotherapy. Sci. Transl. Med..

[46] Kawai T., Akira S. (2007). Signaling to NF-κappaB by Toll-like receptors. Trends Mol. Med..

[47] Engelmann C., Habtesion A., Hassan M., Kerbert A.J., Hammerich L., Novelli S., Fidaleo M., Philips A., Davies N., Ferreira-Gonzalez S. (2022). Combination of G-CSF and a TLR4 inhibitor reduce inflammation and promote regeneration in a mouse model of ACLF. J. Hepatol..

[48] Yu J., Zhu C., Wang X., Kim K., Bartolome A., Dongiovanni P., Yates K.P., Valenti L., Carrer M., Sadowski T. (2021). Hepatocyte TLR4 triggers inter-hepatocyte Jagged1/Notch signaling to determine NASH-induced fibrosis. Sci. Transl. Med..

[49] Di Naso F.C., Porto R.R., Fillmann H.S., Maggioni L., Padoin A.V., Ramos R.J., Mottin C.C., Bittencourt A., Marroni N.A., de Bittencourt P.I. (2015). Obesity depresses the anti-inflammatory HSP70 pathway, contributing to NAFLD progression. Obesity.

[50] Jiang J., Shi Y., Shan Z., Yang L., Wang X., Shi L. (2012). Bioaccumulation, oxidative stress and HSP70 expression in Cyprinus carpio L. exposed to microcystin-LR under laboratory conditions. Comp. Biochem. Physiol. C Toxicol. Pharmacol..

[51] Ji Y., Lu G., Chen G., Huang B., Zhang X., Shen K., Wu S. (2011). Microcystin-LR induces apoptosis via NF-κappaB/iNOS pathway in INS-1 cells. Int. J. Mol. Sci..

[52] He Y., Hara H., Nunez G. (2016). Mechanism and Regulation of NLRP3 Inflammasome Activation. Trends Biochem. Sci..

[53] Lad A., Hunyadi J., Connolly J., Breidenbach J.D., Khalaf F.K., Dube P., Zhang S., Kleinhenz A.L., Baliu-Rodriguez D., Isailovic D. (2022). Antioxidant Therapy Significantly Attenuates Hepatotoxicity following Low Dose Exposure to Microcystin-LR in a Murine Model of Diet-Induced Non-Alcoholic Fatty Liver Disease. Antioxidants.

[54] Lad A., Su R.C., Breidenbach J.D., Stemmer P.M., Carruthers N.J., Sanchez N.K., Khalaf F.K., Zhang S., Kleinhenz A.L., Dube P. (2019). Chronic Low Dose Oral Exposure to Microcystin-LR Exacerbates Hepatic Injury in a Murine Model of Non-Alcoholic Fatty Liver Disease. Toxins.

[55] Zhao Y., Xue Q., Su X., Xie L., Yan Y., Wang L., Steinman A.D. (2016). First Identification of the Toxicity of Microcystins on Pancreatic Islet Function in Humans and the Involved Potential Biomarkers. Environ. Sci. Technol..

[56] Black A.T., Hayden P.J., Casillas R.P., Heck D.E., Gerecke D.R., Sinko P.J., Laskin D.L., Laskin J.D. (2011). Regulation of Hsp27 and Hsp70 expression in human and mouse skin construct models by caveolae following exposure to the model sulfur mustard vesicant, 2-chloroethyl ethyl sulfide. Toxicol. Appl. Pharmacol..

[57] Kuper C., Beck F.X., Neuhofer W. (2012). Toll-like receptor 4 activates NF-κappaB and MAP kinase pathways to regulate expression of proinflammatory COX-2 in renal medullary collecting duct cells. Am. J. Physiol. Renal. Physiol..

[58] Galloway E., Shin T., Huber N., Eismann T., Kuboki S., Schuster R., Blanchard J., Wong H.R., Lentsch A.B. (2008). Activation of hepatocytes by extracellular heat shock protein 72. Am. J. Physiol. Cell Physiol..

[59] He W.T., Wan H., Hu L., Chen P., Wang X., Huang Z., Yang Z.H., Zhong C.Q., Han J. (2015). Gasdermin D is an executor of pyroptosis and required for interleukin-1beta secretion. Cell Res..

[60] Kaiser W.J., Sridharan H., Huang C., Mandal P., Upton J.W., Gough P.J., Sehon C.A., Marquis R.W., Bertin J., Mocarski E.S. (2013). Toll-like receptor 3-mediated necrosis via TRIF, RIP3, MLKL. J. Biol. Chem..

[61] Liu H., Zhang X., Zhang S., Huang H., Wu J., Wang Y., Yuan L., Liu C., Zeng X., Cheng X. (2018). Oxidative Stress Mediates Microcystin-LR-Induced Endoplasmic Reticulum Stress and Autophagy in KK-1 Cells and C57BL/6 Mice Ovaries. Front. Physiol..

[62] Chatterjee S., Ganini D., Tokar E.J., Kumar A., Das S., Corbett J., Kadiiska M.B., Waalkes M.P., Diehl A.M., Mason R.P. (2013). Leptin is key to peroxynitrite-mediated oxidative stress and Kupffer cell activation in experimental non-alcoholic steatohepatitis. J. Hepatol..

[63] Jiang M., He J., Sun Y., Dong X., Yao J., Gu H., Liu L. (2021). Leptin Induced TLR4 Expression via the JAK2-STAT3 Pathway in Obesity-Related Osteoarthritis. Oxid. Med. Cell Longev..

[64] Mondal A., Bose D., Saha P., Sarkar S., Seth R., Kimono D., Albadrani M., Nagarkatti M., Nagarkatti P., Chatterjee S. (2020). Lipocalin 2 induces neuroinflammation and blood-brain barrier dysfunction through liver-brain axis in murine model of nonalcoholic steatohepatitis. J. Neuroinflamm..

[65] Seth R.K., Das S., Dattaroy D., Chandrashekaran V., Alhasson F., Michelotti G., Nagarkatti M., Nagarkatti P., Diehl A.M., Bell P.D. (2017). TRPV4 activation of endothelial nitric oxide synthase resists nonalcoholic fatty liver disease by blocking CYP2E1-mediated redox toxicity. Free Radic. Biol. Med..

[66] Chandrashekaran V., Seth R.K., Dattaroy D., Alhasson F., Ziolenka J., Carson J., Berger F.G., Kalyanaraman B., Diehl A.M., Chatterjee S. (2017). HMGB1-RAGE pathway drives peroxynitrite signaling-induced IBD-like inflammation in murine nonalcoholic fatty liver disease. Redox. Biol..

[67] Alhasson F., Seth R.K., Sarkar S., Kimono D.A., Albadrani M.S., Dattaroy D., Chandrashekaran V., Scott G.I., Raychoudhury S., Nagarkatti M. (2018). High circulatory leptin mediated NOX-2-peroxynitrite-miR21 axis activate mesangial cells and promotes renal inflammatory pathology in nonalcoholic fatty liver disease. Redox. Biol..

[68] Szabo G., Petrasek J. (2015). Inflammasome activation and function in liver disease. Nat. Rev. Gastroenterol. Hepatol..

[69] Wan X., Xu C., Yu C., Li Y. (2016). Role of NLRP3 Inflammasome in the Progression of NAFLD to NASH. Can. J. Gastroenterol. Hepatol..

[70] Wree A., Eguchi A., McGeough M.D., Pena C.A., Johnson C.D., Canbay A., Hoffman H.M., Feldstein A.E. (2014). NLRP3 inflammasome activation results in hepatocyte pyroptosis, liver inflammation, and fibrosis in mice. Hepatology.

[71] Yang S.J., Lim Y. (2014). Resveratrol ameliorates hepatic metaflammation and inhibits NLRP3 inflammasome activation. Metabolism.

[72] Sarkar S., Saha P., Seth R.K., Mondal A., Bose D., Kimono D., Albadrani M., Mukherjee A., Porter D.E., Scott G.I. (2020). Higher intestinal and circulatory lactate associated NOX2 activation leads to an ectopic fibrotic pathology following microcystin co-exposure in murine fatty liver disease. Comp. Biochem. Physiol. C Toxicol. Pharmacol..

[73] Fink S.L., Cookson B.T. (2005). Apoptosis, pyroptosis, and necrosis: Mechanistic description of dead and dying eukaryotic cells. Infect. Immun..

[74] Weiss W.A., Edelman I., Culbertson M.R., Friedberg E.C. (1987). Physiological levels of normal tRNA(CAGGln) can effect partial suppression of amber mutations in the yeast Saccharomyces cerevisiae. Proc. Natl. Acad. Sci. USA.

[75] Conos S.A., Chen K.W., De Nardo D., Hara H., Whitehead L., Nunez G., Masters S.L., Murphy J.M., Schroder K., Vaux D.L. (2017). Active MLKL triggers the NLRP3 inflammasome in a cell-intrinsic manner. Proc. Natl. Acad. Sci. USA.

[76] Johnston A.N., Wang Z. (2020). HSP70 promotes MLKL polymerization and necroptosis. Mol. Cell Oncol..

[77] Nasiri-Ansari N., Nikolopoulou C., Papoutsi K., Kyrou I., Mantzoros C.S., Kyriakopoulos G., Chatzigeorgiou A., Kalotychou V., Randeva M.S., Chatha K. (2021). Empagliflozin Attenuates Non-Alcoholic Fatty Liver Disease (NAFLD) in High Fat Diet Fed ApoE((-/-)) Mice by Activating Autophagy and Reducing ER Stress and Apoptosis. Int. J. Mol. Sci..

[78] Biasizzo M., Kopitar-Jerala N. (2020). Interplay Between NLRP3 Inflammasome and Autophagy. Front. Immunol..

[79] Tao Y., Wang N., Qiu T., Sun X. (2020). The Role of Autophagy and NLRP3 Inflammasome in Liver Fibrosis. Biomed. Res. Int..

[80] Yang Q., Shu F., Gong J., Ding P., Cheng R., Li J., Tong R., Ding L., Sun H., Huang W. (2020). Sweroside ameliorates NAFLD in high-fat diet induced obese mice through the regulation of lipid metabolism and inflammatory response. J. Ethnopharmacol..

[81] Mridha A.R., Wree A., Robertson A.A.B., Yeh M.M., Johnson C.D., Van Rooyen D.M., Haczeyni F., Teoh N.C., Savard C., Ioannou G.N. (2017). NLRP3 inflammasome blockade reduces liver inflammation and fibrosis in experimental NASH in mice. J. Hepatol..

[82] Garcia-Tsao G., Bosch J., Kayali Z., Harrison S.A., Abdelmalek M.F., Lawitz E., Satapathy S.K., Ghabril M., Shiffman M.L., Younes Z.H. (2020). Randomized placebo-controlled trial of emricasan for non-alcoholic steatohepatitis-related cirrhosis with severe portal hypertension. J. Hepatol..

[83] Rivera C.A., Adegboyega P., van Rooijen N., Tagalicud A., Allman M., Wallace M. (2007). Toll-like receptor-4 signaling and Kupffer cells play pivotal roles in the pathogenesis of non-alcoholic steatohepatitis. J. Hepatol..

[84] Fawell J.K., Mitchell R.E., Everett D.J., Hill R.E. (1999). The toxicity of cyanobacterial toxins in the mouse: I microcystin-LR. Hum. Exp. Toxicol..

[85] Yan Z., Wei H., Ren C., Yuan S., Fu H., Lv Y., Zhu Y., Zhang T. (2015). Gene expression of Hsps in normal and abnormal embryonic development of mouse hindlimbs. Hum. Exp. Toxicol..

[86] Bouhy D., Juneja M., Katona I., Holmgren A., Asselbergh B., De Winter V., Hochepied T., Goossens S., Haigh J.J., Libert C. (2018). A knock-in/knock-out mouse model of HSPB8-associated distal hereditary motor neuropathy and myopathy reveals toxic gain-of-function of mutant Hspb8. Acta Neuropathol..

[87] Liu G., Friggeri A., Yang Y., Park Y.J., Tsuruta Y., Abraham E. (2009). miR-147, a microRNA that is induced upon Toll-like receptor stimulation, regulates murine macrophage inflammatory responses. Proc. Natl. Acad. Sci. USA.

