# Supplementary material for: Hepatic NLRP3-Derived Hsp70 Binding to TLR4 Mediates MASLD to MASH Progression upon Inhibition of PP2A by Harmful Algal Bloom Toxin Microcystin, a Second Hit
Source: Int J Mol Sci. 2023 Nov 15;24(22):16354. doi: 10.3390/ijms242216354 (PMC10671242; doi:10.3390/ijms242216354)
Supplement: Supplementary file 1 [file ijms-24-16354-s001.zip › ijms-2687438-supplementary.pdf]

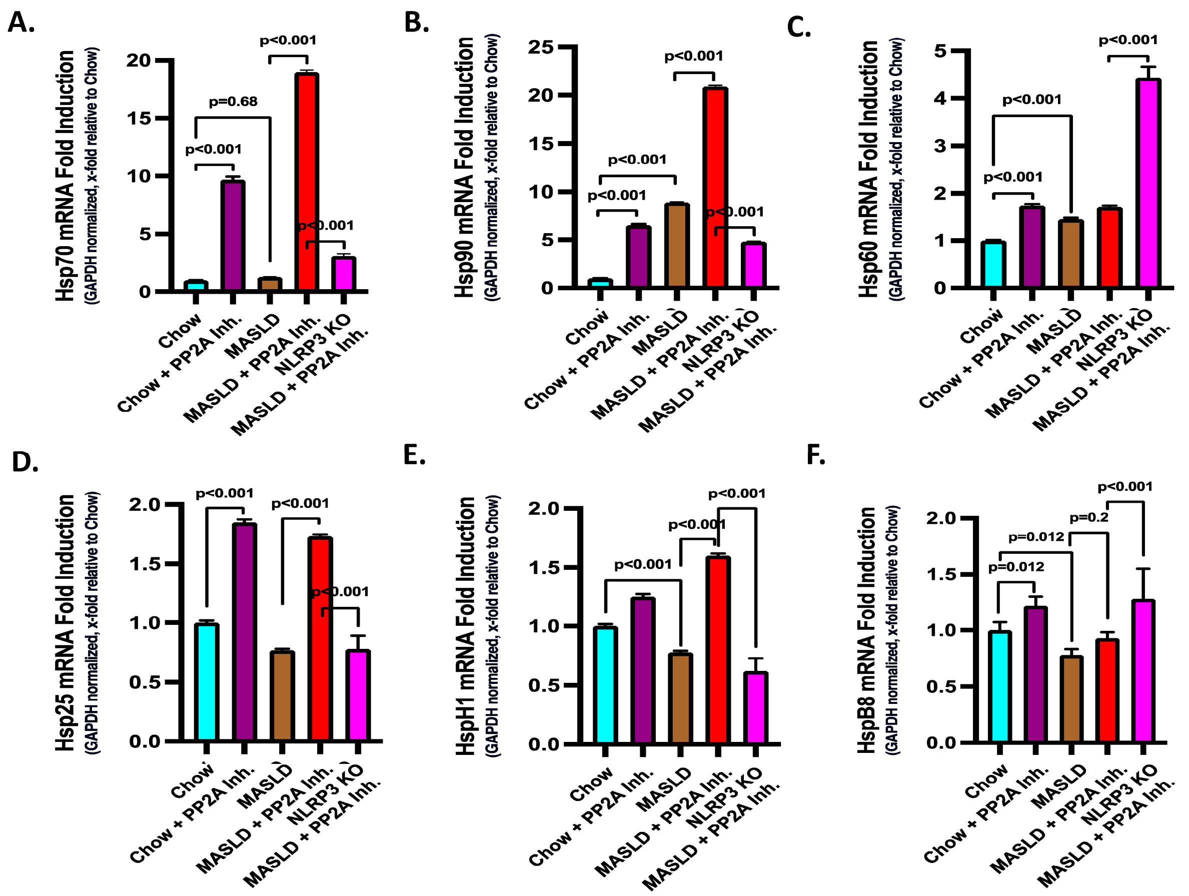

Figure S1. mRNA level expression screening of pertinent Heat shock proteins. A-F: mRNA expression analyses of Hsp70, Hsp90, Hsp60, Hsp25, HspH1, HspB8 in the liver lysates of Chow, Chow + PP2A inhibitor, MASLD, MASLD + PP2A inhibitor and NLRP3 KO MASLD + PP2A inhibitor mouse samples. mRNA expression was assessed by quantitative real-time PCR, and expression was normalized against GAPDH expression and fold increase calculated against the Chow group. All the p-values were determined through one-way ANOVA with the significance level set at $p < 0.05$ (n=3 in biological replicates).
